# The impact of facial abnormalities and their spatial position on perception of cuteness and attractiveness of infant faces

**DOI:** 10.1371/journal.pone.0180499

**Published:** 2017-07-27

**Authors:** Jennifer Lewis, Debi Roberson, Tom Foulsham

**Affiliations:** Department of Psychology, University of Essex, Colchester, Essex, United Kingdom; Medical University of South Carolina, UNITED STATES

## Abstract

Research has demonstrated that how “cute” an infant is perceived to be has consequences for caregiving. Infants with facial abnormalities receive lower ratings of cuteness, but relatively little is known about how different abnormalities and their location affect these aesthetic judgements. The objective of the current study was to compare the impact of different abnormalities on the perception of infant faces, while controlling for infant identity. In two experiments, adult participants gave ratings of cuteness and attractiveness in response to face images that had been edited to introduce common facial abnormalities. Stimulus faces displayed either a haemangioma (a small, benign birth mark), strabismus (an abnormal alignment of the eyes) or a cleft lip (an abnormal opening in the upper lip). In Experiment 1, haemangioma had less of a detrimental effect on ratings than the more severe abnormalities. In Experiment 2, we manipulated the position of a haemangioma on the face. We found small but robust effects of this position, with abnormalities in the top and on the left of the face receiving lower cuteness ratings. This is consistent with previous research showing that people attend more to the top of the face (particularly the eyes) and to the left hemifield.

## Introduction

An infant’s appearance has important effects on the way it is judged [1–4]. It has been shown that infants who are perceived as more attractive are more likely to elicit care-giving behaviours from adults [5–12] and they may lead to a greater level of reward circuitry activation in the brains of adult observers [13]. Despite this, there is much to be uncovered about what makes particular infant faces more likeable and how robust this is across observers. This is particularly important for the small percentage of infants born with craniofacial abnormalities, such as a cleft lip (an opening in the upper lip which occurs when the tissues of the face do not join fully) or a haemangioma (a small red birth mark). The current studies are aimed at investigating these abnormalities, their impact on preference judgements, and the role of their spatial position on the face.

### Defining facial attractiveness

Humans show a high level of agreement when asked to judge the attractiveness of an adult face. These judgements have been assumed to reflect a common aesthetic response which is particularly associated with sexual attractiveness and the process of selecting a mate from the opposite sex. Many studies have investigated the specific characteristics which attractive faces have in common. For example, Rhodes [14] summarises the evidence that facial symmetry, averageness and sexual dimorphism are independent components of attractiveness. On average, both men and women prefer faces which are symmetrical, closer to the population average and more strongly masculine or feminine. It has been proposed that these preferences are evolutionarily adaptive when choosing mates because they signal physical or genetic health (or may have done in our evolutionary history).

Abnormalities such as a cleft lip have an impact on perceived attractiveness in adults and this has been linked to dimensions such as symmetry [15]. By this account, the psychosocial effects of even benign abnormalities are partly due to their effect on the components of facial attractiveness discussed above. However, before discussing effects of abnormality on infants we must consider what makes an *infant* face aesthetically pleasing—typically described as “cuteness”.

### Defining cuteness

The Merriam-Webster dictionary defines the adjective “cute” as “attractive or pretty especially in a childish, youthful, or delicate way” [16]. The common usage of the term “cuteness” therefore indicates a specific type of attractiveness associated with children and youthful features. As with adult faces, humans show a considerable level of agreement when asked to judge the cuteness of an infant face [1–4, 17]. Moreover, subjective cuteness ratings have been reported to correlate with more objective behaviours, such as the willingness to press a key to prolong viewing time [17], the reported willingness to adopt the infant [8, 18] and even the outcomes of infants in hospital [5].

Cuteness is also defined by a particular configuration of facial features which is theorised to trigger an innate caregiving mechanism (the *Kindchenschema* [7,19]). These features include a large forehead, large eyes, chubby cheeks and facial features set low in the face. Several studies have confirmed that the presence of these features is associated with explicit judgements. For example, Glocker et al. [7] manipulated images of the same infant to increase the presence of these features (e.g., by increasing the size of the forehead and eyes). The manipulated features correlated with the ratings of cuteness given by adult observers. Parsons et al. [20] also demonstrated that the degree of infantile features correlated with both explicit attractiveness ratings and the willingness of observers to press a key in order to increase the viewing duration.

Cuteness in infants, therefore, is associated with particular facial characteristics that are distinct from adult attractiveness and linked to different adaptive behaviours associated with caregiving rather than mate selection. Some adults may be “baby-faced” and judged as more naïve, trustworthy and helpless [21, 22], but the cuteness of individuals as infants does not predict the attractiveness ratings of the same individuals as adults [23]. In the present study, we use subjective ratings of cuteness as a measure of aesthetic preference for particular infant faces, which is proposed to reflect the Kindchenschema-triggering features described above. This is also the approach followed by most recent research investigating infant faces [7, 8, 17, 20], quantifying what Spregelmeyer et al [17] describe as the “aesthetic salience” of a face.

Previous research using infant faces has used either the specific term “cute” or the more general term “attractive” as instructions to solicit aesthetic judgements [e.g., 6, 7, 20]. However, to our knowledge no study has compared both labels within the same set of faces. In the present study, we used both the term “cute” and the term “attractive” when instructing participants to rate images, but our assumption was that in infants they would elicit similar ratings.

### The impact of abnormalities

Given the widespread influence of an infant’s cuteness on judgements and behaviours, it is important to consider the impact of abnormalities on the face. Three of the most common and noticeable facial abnormalities affecting infants are haemangioma, strabismus and cleft-lip. These have a range of causes and consequences, and aesthetic judgements are an important aspect of decisions about treatment and surgery. Haemangioma are benign “strawberry” marks which are seen in about 10% of 1-year old infants, most frequently occurring on the face or neck [24]. Strabismus, an abnormal alignment of the eyes, is present in around 2–5% of children [25], where it has both functional and psychosocial consequences [26]. A cleft-lip occurs much less often, when the tissues of the face do not fuse correctly during development.

Facial abnormalities have been found to cause a significant reduction in cuteness ratings [27]. A recent study by Parsons et al [20] also found that viewing images of infants with a cleft-lip resulted in a diminished level of activation in the medial orbitofrontal cortex of observers. This area is associated with reward activation and has been specifically implicated as being part of the neural mechanism mediating the caregiving response [28]. Adults and children with strabismus are perceived as less attractive and judged to be less likely to find a partner or be invited to a party [29]. However, because of the variety of tasks, measures and age groups used in previous research, the effect of different types of infant facial abnormality on judgements is not clear.

### Spatial selection in faces

One factor that might change the effect of an abnormality is its position on the face. Research investigating eye movements during face viewing has revealed that regardless of task demands (e.g. free viewing or judging age, expression, gender or identity) Western Caucasian participants spend the greatest proportion of dwell time and make the greatest number of fixations to the eye region [30–35]. This increased attention to the eye region, (which may also include the eyebrows and other regions near the eyes) results in more fixations on the top half of the face. Eastern Asian individuals have been shown to spend a greater proportion of dwell time on the nose region compared with the eye region, however, they also spend most of their time looking at the core features of the face, and are less likely to look at the mouth [36].

As well as a bias towards looking at the top half of the face, individuals also show a left hemiface bias. This bias describes the tendency of individuals to use information from the left hemiface (the side of a face that is on the *observer’s* left as they look at it) when making judgments about factors such as gender, expression and attractiveness [37,38]. In eye-tracking experiments, this perceptual bias is reflected in a left-gaze-bias: a tendency for individuals to make their first fixation and spend a greater proportion of dwell time in the left hemiface during judgment tasks [39–42]. The left bias for face processing may also reflect fMRI and ERP evidence that areas associated with face processing (such as the fusiform face area) are more dominant in the right hemisphere during face processing [43–45].

Given that some areas of the face receive more attention than others, it is possible that abnormalities in these parts of the face will be particularly noticeable. This could cause a greater reduction in preference ratings. However, if abnormalities are quite salient then their visibility will be high, and they may attract attention regardless of their location. Recent evidence for this comes from Meyer-Marcotty et al [15], who found that individuals look more at the mouth and nose region of individuals with a cleft-lip. If abnormalities attract attention wherever they occur, they may cause a uniform reduction in cuteness ratings.

### The present study

The present study aimed to measure the effect of different facial abnormalities, and in particular, their spatial location, on aesthetic evaluations. In Experiment 1, we began by comparing the effect of different abnormalities, while in Experiments 2a and 2b, we concentrated on manipulating the position of a single common abnormality (a haemangioma). In each case, we predicted that judgements would be affected by the presence of an abnormality. Based on previous research into spatial biases in face perception, we predicted that abnormalities on the left and top half of the face (in Experiments 2a and 2b) would be more detrimental for aesthetic ratings. Finally, given that relatively few studies have examined the perception of infant cuteness, we asked whether “cuteness” and “attractiveness” instructions elicited judgements which were reliable across raters, correlated and affected by abnormalities in the same way.

## Experiment 1

### Method

#### Ethics statement

Ethical approval was received through the University of Essex Ethics Committee and written, informed consent was obtained from each participant prior to the start of the study.

#### Participants

One hundred and sixty-seven participants from the USA completed the study (83 females). Participants had a mean age of 31.0 years and took part online in exchange for payment. Participants were randomly allocated to either the “cuteness” or “attractiveness” instructions.

#### Stimuli

The stimuli were created from a subset of the photographs taken and described by Hildebrandt and Fitzgerald [46], who obtained parental consent to use these images. From this original set, 49 images of different individual infants were selected for use in the present experiment. No particular criteria were used to select these faces, but stimuli were excluded where hair obscured areas of the face or photographic artefacts would have made editing the images more difficult. The infants ranged from 3 months to 13 months old and all had a neutral facial expression. These images were modified using the software GIMP in order to remove any background, straighten and crop the images to a standard size.

**Fig 1:**
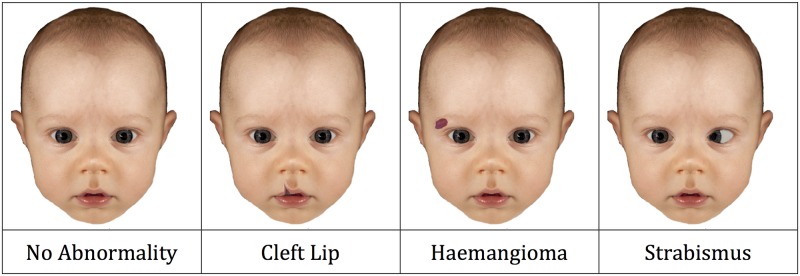
Example of the four conditions used in Experiment 1. For copyright reasons, this figure is for illustration only and not the actual stimuli. Altered from [53].

Four versions of each image were created: an original, un-modified image and one each with haemangioma, strabismus and cleft lip. Standard examples of haemangioma were taken from an online image search and carefully pasted and blended into the images. Several different examples of haemangioma were used, and these were resized and rotated slightly to ensure variation across infants and obtain a natural appearance. To create the strabismus images, one eye from each image was rotated inwards by editing the position of the pupil, iris and sclera, to create a set of images with esotropia. The eye rotated inwards (left or right) was counterbalanced across the 49 images. To create the cleft lip images, standard examples of this abnormality were taken from images found online and pasted onto the original face image. The size and colour of the cleft lip mouth was adjusted in order to blend into the original images and create a natural appearance, and approximately half of the mouths were flipped horizontally in order to ensure that horizontal location was counterbalanced between infants. In numerous experiments with these stimuli, naïve participants have been unable to notice that the faces have been digitally altered. Fig 1 illustrates all 4 types of face (for copyright reasons this figure is for illustration only and not the actual stimuli). Stimuli are available from the authors on request.

In order to counterbalance identity and abnormality across participants, the images were distributed across four sets. Each participant saw each individual infant only once, but across participants every infant appeared in all four abnormality conditions. Each experiment therefore consisted of all 49 infants, with 12 or 13 examples of unmodified, haemangioma, strabismus and cleft lip faces.

#### Procedure

The experiment was conducted using the web-based survey system Qualtrics. Prior to the task, participants were given written instructions telling them that their task was to judge either “how cute” or “how attractive” they thought each face was. Participants then completed the ratings questionnaire at their own pace. On each page, one of the 49 images was presented with the question “How cute is the infant in the picture?” in the cuteness task, and, “How attractive is the infant in the picture?” in the attractiveness task. Underneath each image was a 7-point Likert scale which ranged from “Not very cute” to “Very cute”, with a central anchor of “Average cuteness” (or corresponding wording in the attractiveness condition). To indicate their decision, the participant checked a value from one to seven using the mouse. There was no time limit, and faces were presented in a randomized order.

### Results

The results consisted of ratings on a 1–7 scale. These were analysed to address research questions regarding the reliability and similarity of cuteness and attractiveness ratings, and the effect of different abnormalities. The ratings data from Experiments 1 and 2 are included in S1 Dataset. Stimuli are available on request.

#### Reliability and the relationship between cuteness and attractiveness judgements

In this experiment, participants rated infant faces for *either* cuteness or attractiveness. Each particular face was rated by at least 16 participants. Within the unmodified faces, inter-rater reliability for cuteness judgments was good (Cronbach’s alphas from 0.77 to 0.90 across image sets). Judgements in response to attractiveness instructions were also reliable across raters (alphas from 0.76 to 0.93). The ratings of four participants were excluded at this point because they gave the same response to every face.

We repeated the reliability analysis with the faces with abnormalities. With cuteness instructions, ratings of faces with abnormalities showed adequate reliability (alphas from 0.62 to 0.81). Judgements of attractiveness also showed good inter-rater reliability (alphas from 0.61 to 0.87). Across all image sets, there was no evidence that ratings to faces with particular abnormalities were less reliable.

Next, an average cuteness rating and an average attractiveness rating was calculated for each face, across all judges. There was a strong, positive correlation between the mean cuteness and attractiveness rating received by unmodified faces, *r* = .837, *N* = 49, *p* < .001. The correlations between the mean cuteness and attractiveness ratings received by faces with abnormalities were also positive and statistically significant (haemangioma: *r* = .77; cleft lip: *r* = .61; strabismus: *r* = .46; all *N*s = 49, *p*s < .001). Thus, in general, infants who were given high ratings with cuteness instructions were also given high ratings with attractiveness instructions. However, the two ratings were less well correlated in faces with abnormalities, and especially in those with strabismus. For this reason, in the following analysis we consider the impact of abnormalities on both cuteness and attractiveness.

#### Effects of different abnormalities

One of the advantages of our design was that the same individual infants appeared in each abnormality condition, but counterbalanced across different participants. To examine the effects of abnormalities on cuteness and attractiveness ratings we used linear mixed effects (LME) models, which are increasingly preferred over traditional ANOVA designs and which allow both items and participants to be modelled as random effects. Data are modelled at the level of individual observations. We followed a model-building approach by adding fixed, categorical factors representing the type of abnormality (no abnormality, haemangioma, cleft lip and strabismus) and the task instructions (cuteness rating and attractiveness rating) and comparing nested models using likelihood ratio tests [47]. Model fitting was accomplished using the lme4 package in R, and fixed effects associated with a *t* value greater than 2 were considered statistically significant. Follow-up comparisons were carried out using the lsmeans package in R and interactions were decomposed with additional LMEs to investigate simple effects.

We began by testing the fixed effect of abnormality, with random effects of participant and face. This model outperformed a null model with a fixed intercept (χ^2^(3) = 2730, *p* < .001). Thus, the presence of an abnormality made a difference to the cuteness and attractiveness ratings. Model estimates demonstrated that faces with all three types of abnormality received ratings more than one point lower, on average, than those with no abnormality (Haemangioma: β = -1.09, SE = 0.03, *t* = 33.2; Cleft lip: β = -1.58, SE = 0.03, *t* = 48.1; Strabismus: β = -1.66, SE = 0.03, *t* = 50.6). Pairwise comparisons revealed that faces with haemangioma (*M* = 3.27, 95%CI = [3.08, 3.46]) received lower ratings than unmodified faces (*M* = 4.36, 95%CI = [4.17, 4.56]), but higher ratings than those with cleft lip (*M* = 2.78, 95%CI = [2.59, 2.97]) or strabismus (*M* = 2.70, 95%CI = [2.51, 2.89]). All of the pairwise differences were statistically significant (*t*s>14, *p*s < .001, with Tukey adjustment for multiple comparisons), with the exception of the contrast between faces with cleft lip and strabismus, which failed to reach significance (*t* = 2.4, *p* = .07).

**Fig 2:**
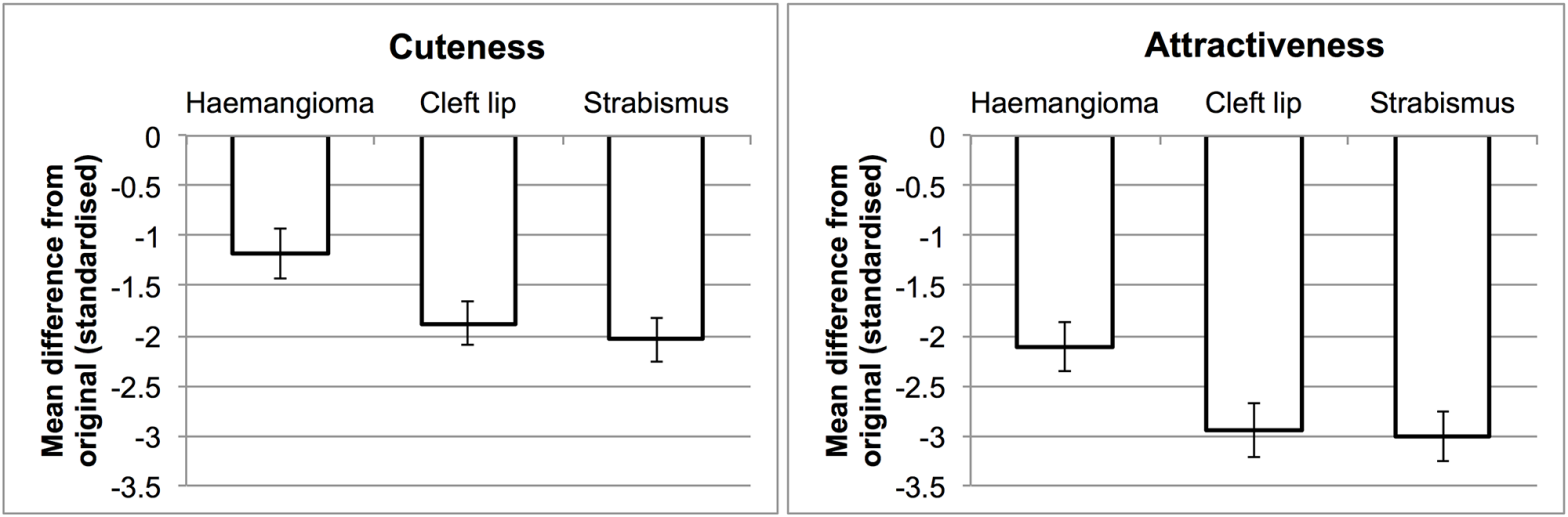
The effect of each type of abnormality on mean ratings of cuteness (left) and attractiveness (right). Bars show the mean difference from the no abnormality condition, calculated within each item (face), and standardised by the standard deviation of this condition. Error bars show the 95% confidence intervals around this difference.

Next, we estimated a model with the additional fixed factor of task instructions, as well as the interaction between task instructions and abnormality condition. This further improved the model fit (χ^2^(4) = 63.1, *p* < .001), indicating that the particular instructions (using the label cuteness or attractiveness) made a difference to ratings. By itself, task was not a reliable predictor (β = -0.17, SE = 0.17, *t* = 1.0, with attractiveness as the reference level), but the interaction suggested that cuteness and attractiveness ratings were differently affected by abnormalities. This was probed with follow-up LME models for cuteness and attractiveness rating separately. Table 1 shows the resulting LME models, and Fig 2 shows the standardised effect of each condition compared to the original infant faces. Although the pattern is similar in both rating tasks, abnormalities had a smaller detrimental effect on cuteness ratings than on attractiveness ratings.

**Table 1:** Summary of linear mixed effects models predicting ratings of faces with different abnormalities.

	Fixed effect / level	β	*SE*	*t*
Cuteness	Intercept	4.27	0.15	29.2 *
	Haemangioma	-0.84	0.05	16.7 *
	Cleft lip	-1.33	0.05	26.6 *
	Strabismus	-1.45	0.05	29.0 *
Attractiveness	Intercept	4.44	0.11	39.7 *
	Haemangioma	-1.28	0.04	29.6 *
	Cleft lip	-1.78	0.04	41.1 *
	Strabismus	-1.83	0.04	42.2 *

Estimates show the contrast of each level with the no abnormality condition. Predictors associated with t-values greater than 2 are considered statistically significant (*).

### Discussion

The perceived cuteness of an infant’s face can have important consequences. Experiment 1 therefore sought robust estimates of how this perception is altered in cases of facial abnormality. Although previous research has shown that abnormalities affect both explicit judgements of cuteness and reward-related brain activity, few studies have controlled for the particular infant face or been able to compare different types of abnormality.

Our results confirm that three common facial abnormalities (haemangioma, strabismus and cleft lip) cause the very same infants to be rated as less cute. Haemangioma had the smallest negative impact on ratings. This is likely because strabismus and cleft lip are seen as more severe abnormalities, with functional consequences. Haemangioma are also more common than the other abnormalities, meaning that participants would have been more familiar with them. It is also the case that strabismus and cleft lip abnormalities are associated with the core features of the face, which most observers spend most of the time looking at, while haemangioma are both more benign and affect peripheral areas of the face.

The findings also showed a close correlation between ratings given to particular infants in response to “cuteness” and “attractiveness” instructions. Under these conditions, participants seemed to treat these terms in the same way, and different types of abnormality had a similar effect on both types of rating. However, it is interesting to note that all abnormalities had a larger standardized effect on attractiveness rating than cuteness rating. It may be that this is because such abnormalities have a larger impact on characteristics typically seen as attractive in adults (i.e., averageness and symmetry) than on features seen as cute (Kindchenschema features, which are largely unaffected by these abnormalities). In future research it would be interesting to see how abnormalities affect ratings of adult faces, where attractiveness and cuteness may be more separable.

The abnormalities used in this experiment affect different parts of the face, but they also vary in their severity and implications. In Experiment 2 we hold the severity of the abnormality constant while manipulating its location.

## Experiment 2

### Method

#### Ethics statement

Ethical approval was received through the University of Essex Ethics Committee and informed consent was obtained from each participant prior to the start of the study via a written consent form (Experiment 2(a)) or an online form (Experiment 2(b)).

#### Participants

Experiment 2 drew on two different samples of participants, in order to collect a range of different observer judgements. In Experiment 2(a), 59 student volunteers took part at the University of Essex in exchange for payment or course credit. There were 37 females and participants had an average age of 22.6 years. In Experiment 2(b), 83 participants from the USA completed the study online in exchange for payment. There were 41 females and the mean age was 33.5 years.

#### Stimuli

**Fig 3:**
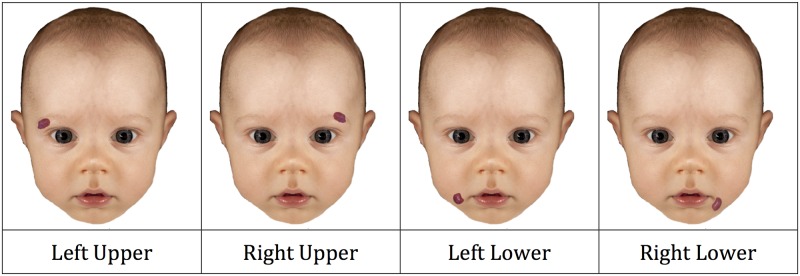
Example of the four haemangioma categories used in Experiment 2. For copyright reasons, this figure is for illustration only and not the actual stimuli. Altered from [53].

The stimuli were drawn from the same set as in Experiment 1, but for counterbalancing an even number of 48 individual infant faces were used. Due to experimenter error, one image was not presented and thus analyses are based on 47 faces. The original face images were used for the unmodified condition, and additional versions were created using photo-editing software (GIMP) in order to manipulate the location of a haemangioma. Half of the faces were given an abnormality in the upper half of the face (near the eyes), while the other half were given one in the lower half of the face (near the mouth). In each case, two versions were made: haemangioma on the left and haemangioma on the right, with the shape and size kept constant. Fig 3 illustrates all four of the resulting location conditions.

As in Experiment 1, each participant saw each individual infant only once per task, but images were counterbalanced across sets so that across the experiment each infant appeared in all conditions. Each experimental task therefore consisted of 47 ratings trials comprising approximately 15 unmodified infant faces and 8 from each of the four haemangioma conditions.

#### Procedure

The ratings procedure was administered via Qualtrics, in exactly the same way as in Experiment 1. However, in this experiment we asked participants to complete the ratings tasks with *both* “cuteness” and “attractiveness” instructions. These instructions were given in separate blocks, in a counterbalanced order, and participants were only informed about the second set of instructions after they had completed the first block. In Experiment 2(a), participants took part one at a time while seated in a laboratory, where they were given verbal instructions. In Experiment 2(b), participants took part online.

### Results

In experiments 2(a) and 2(b), all participants rated faces for both cuteness and attractiveness, in a counterbalanced order. We therefore looked again at the relationship between these tasks, and their order, before considering the effect of abnormality position.

#### Reliability and the relationship between cuteness and attractiveness judgements

Across the two samples, each particular unmodified face was rated by at least 20 participants in each task. When calculated from the first (“naïve”) set of ratings given by each participant, both scales showed very good inter-rater reliability (Cuteness: Cronbach’s alphas from 0.83 to 0.88 across image sets; Attractiveness: alphas from 0.82 to 0.91). One participant (from Experiment 2a) was excluded because their ratings showed zero variance across faces. The ratings given to faces with haemangioma also demonstrated good inter-rater reliability (all alphas > 0.8 in both cuteness and attractiveness rating).

An average cuteness and an average attractiveness rating was calculated, for each unmodified (no abnormality) face, by averaging across all judges from the first ratings task only. There was a strong positive correlation between these average cuteness and attractiveness ratings, in both experiments (*r* = .75 and *r* = .62, in Experiments 2a and 2b, respectively; both *p*s < .001). There was also a strong correlation between the ratings given in the two experiments (within cuteness ratings, *r* = .67; within attractiveness, *r* = .77; both *p*s < .001). Therefore, participants in the two samples showed significant agreement, and there was also evidence that cuteness and attractiveness instructions were being treated in a similar fashion. Faces which were rated as highly cute were also likely to be rated as highly attractive. The same was true in faces with haemangioma. The correlation between the mean cuteness and attractiveness rating given to a face with a haemangioma was positive, strong and statistically significant (pooled across experiments, *r* = .76, *p* < .001).

Unlike in Experiment 1, participants in Experiments 2(a) and 2(b) rated each face twice, on both cuteness and attractiveness. It was important, therefore, to check whether the order of these judgements made a difference to the way a face was rated. To do so, we also calculated a face’s mean rating by averaging across all judges from the second ratings task. In Experiment 2(a), faces were given significantly higher ratings in the second task (cuteness: *M* = 4.3, *SD* = 0.78; attractiveness: *M* = 4.1, *SD* = 0.77) than in the first (cuteness: *M* = 3.9, *SD* = 0.71; attractiveness: *M* = 3.9, *SD* = 0.70; paired t-test collapsing across scales, *t*(46) = 4.7, *p* < .001). The same trend was seen in cuteness ratings in Experiment 2(b), where the second task led to higher ratings (*M* = 4.7, *SD* = 0.76) than the first (*M* = 4.0, *SD* = 0.82; *t*(46) = 6.4, *p* < .001). Attractiveness ratings in this experiment actually decreased slightly from the first (*M* = 4.2, *SD* = 0.71) to the second task (*M* = 4.0, *SD* = 0.86; *t*(46) = 1.9, *p* = .06).

In sum, asking for ratings of “cuteness” and “attractiveness” yielded highly similar estimates, as shown by the strong correlations. However, as a conservative step, and because of the evidence for order effects when participants saw a face a second time, we included only the first set of ratings provided by each participant in our main analysis.

#### Effects of haemangioma

Together, Experiments 2(a) and 2(b) had 141 participants, and haemangioma presence and position were manipulated across different image sets. As in Experiment 1, we used linear mixed effects (LME) models and the R package lme4 to test the fixed effects of haemangioma across individual observations. We also tested interactions with the fixed effect of rating type (cuteness or attractiveness), in order to see whether any effects of haemangioma varied with these different instructions.

First, we tested the fixed effect of abnormality presence, with random effects of participant and infant. This compares the rating received by faces with a haemagioma to that received by faces with no abnormality, while controlling for the particular infant and rater. This model outperformed a null, intercept-only model (χ^2^(1) = 523.6, *p* < .001). As expected, faces with an abnormality (*M* = 3.22; 95%CI = [3.01, 3.43]) received significantly lower ratings than those without (*M* = 3.88; 95%CI = [3.67, 4.09]); β = -0.66, *SE* = 0.028, *t* = 23.3). Neither the fixed effect of rating type nor the interaction between rating type and abnormality presence significantly improved the model fit (χ^2^(2) = 5.6, *p* = .06) and the presence of abnormalities had a similar effect on both types of rating.

Next, we looked within only those faces with an abnormality, fitting a model with fixed factors for the horizontal location of the haemangioma (left or right hemiface) and the vertical location (top or bottom of the face). These factors made a significant difference to the model (compared to a null model; χ^2^(2) = 9.6, *p* < .01), and both horizontal and vertical factors were reliable predictors (see Table 2 for full model). Again, adding a main effect of rating type and interactions between haemangioma position and rating type did not improve the model fit (χ^2^(3) = 4.9, *p* = .18). This indicates that the effects of haemangioma were equivalent regardless of whether cuteness or attractiveness instructions were used.

**Table 2:** Results from a linear mixed effects model predicting attractiveness/cuteness rating from location of a haemangioma.

Predictor	β	*SE*	*t*
Intercept	3.34	0.128	26.12 *
Horizontal location (left)	0.066	0.029	2.28 *
Vertical location (lower)	-0.276	0.128	2.15 *

Parenthesised locations show the reference level in each case. Predictors associated with t-values greater than 2 are considered statistically significant (*).

**Fig 4:**
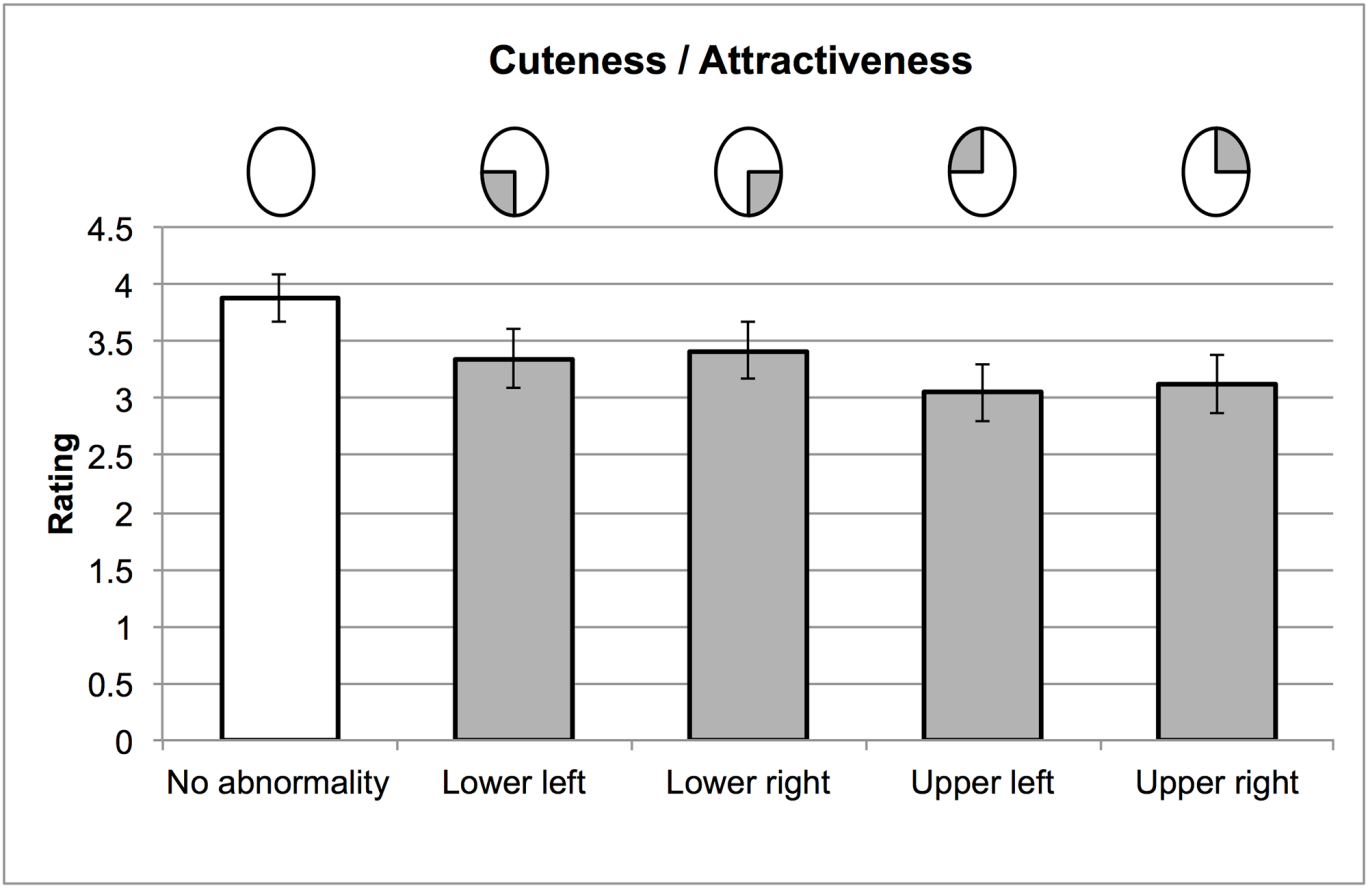
The effect of haemangioma on ratings of cuteness and attractiveness in Experiment 2. Bars show model estimates from the LME models reported in the text, as well as 95% confidence intervals from 1000 bootstrapped simulations.

**Fig 5:**
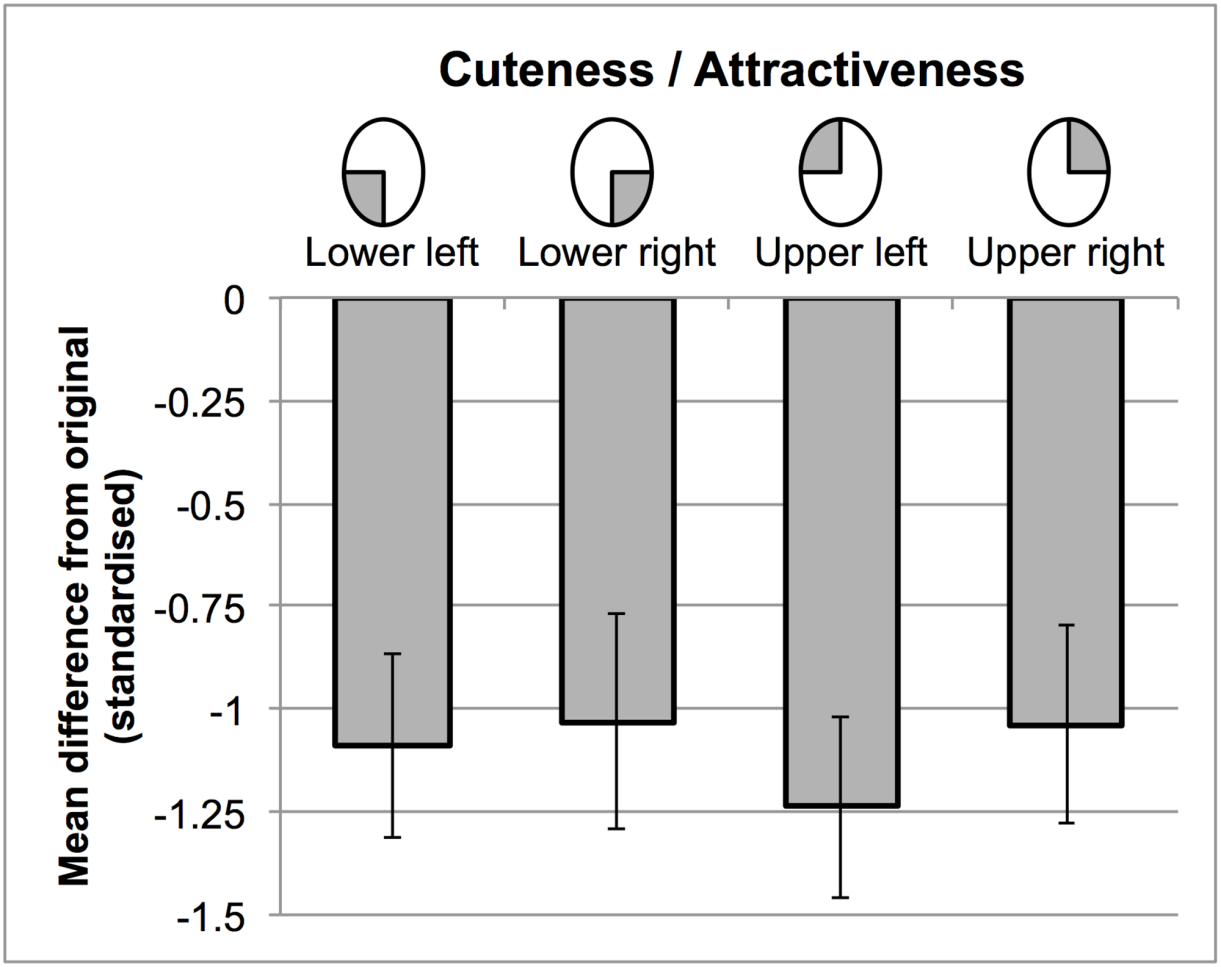
The effect of differently located haemangioma on ratings of cuteness/attractiveness. Bars show the mean difference from the no abnormality condition, across items, and standardised by the standard deviation of this condition. Error bars show the 95% confidence intervals around this difference.

Fig 4 shows estimated ratings for each haemangioma condition, based on the LME models. Fig 5 shows the standardized effect of abnormalities in each location (c.f. Fig 2). Abnormalities in the top half of the face were rated less attractive/cute than those in the bottom half of the face. The effect of horizontal position was smaller, but demonstrated that abnormalities in the left hemiface were more detrimental to preference judgements. Although the effect of horizontal position may have been larger when the abnormality was in the top half of the face, entering the interaction between horizontal and vertical position did not improve the model fit further (χ^2^(1) < 1). Inspection of the marginal means across the two tasks confirmed that–in both cuteness and attractiveness judgements—faces with a haemangioma in the upper left received the lowest ratings, while faces with haemangioma in the lower right received the highest

## General discussion

The way infants are judged can have an important impact on their future wellbeing. Although much is known about the factors that affect attractiveness judgements in adults, far less is known about the evaluation of infant faces. We sought to extend previous research into facial abnormalities in infants by considering their impact on cuteness perception, while controlling for infant identity.

In both experiments, and across three different samples of participants, ratings of cuteness and attractiveness were reliable and consistently reduced by the presence of an abnormality. Previous research has demonstrated some factors such as age and sex which have an effect on perceived cuteness. For example, female infants receive higher average ratings than male infants, and ratings increase from 3 to 11 months of age [46]. Importantly, because the same individual infants appeared in all abnormality conditions, our results cannot be explained by any variability in infant age or sex. Instead, our results demonstrate how the presence and location of abnormalities changes perceived cuteness, which is important given the potential for correcting such abnormalities with surgery or cosmetics. In future research it would be interesting to establish whether abnormalities have a different effect at different ages, but the current stimuli were not varied enough to address this issue.

We also found strong positive correlations between ratings given with different instructions (“cuteness” or “attractiveness”) and little evidence that these two terms were interpreted differently. While this is not surprising, it is helpful when comparing between other studies which have used a range of terms and scales [7,20,23,27].

### Applying objective measures of facial attractiveness

As described in the introduction, both attractiveness in adults and cuteness in infants have been associated with objective measures of particular facial characteristics. How do these characteristics apply to the present results?

In adults, three of the most commonly studied predictors of facial attractiveness are symmetry, averageness and sexual dimorphism. For example, Munoz-Reyes et al [48] measured the facial Fluctuating Asymmetry (FA) in photographs of a large number of women by identifying facial landmarks and comparing the distance between them. The results showed that FA correlated with ratings of attractiveness given by male observers as well as self-ratings from the women themselves. It is clear that the lateralized abnormalities used in the present study would have made the infant faces less symmetrical and lower in averageness (i.e., more distinctive relative to the overall set of faces). However, these objective measures would not have varied between different abnormalities or between haemangioma in different positions on the face. There is some evidence that positive responses to symmetry are adaptive because of a link with health [14], and abnormalities in general may fit this pattern.

However, given that objective cues to attractiveness in adult faces are associated with sexual selection, it is unlikely that these qualities are relevant for adults rating infant faces. Instead, we should consider how abnormalities might affect the Kindchenschema features of round, chubby cheeks, big, low-set eyes and a large forehead. Although participants in our study largely treated “cuteness” and “attractiveness” synonymously, there was some evidence in Experiment 1 that abnormalities have less of an impact on cuteness. This might be because all of the abnormalities affected dimensions such as symmetry, but they did not affect the Kindchenschema. Future research could investigate the interpretation of the two terms in more detail by asking participants to judge both simultaneously (and thus drawing attention to their differentiation), or by determining their association with related constructs.

Importantly, while variations in Kindchenschema might explain why some (unmodified) infant faces received reliably higher ratings than others [7], it seems unlikely that they can explain the effects of abnormality type (and particularly abnormality position). This is because none of the abnormalities changed the overall shape or size of the relevant features and so objective measures such as face width or forehead or eye size were not affected. Future research could investigate whether other abnormalities or positions might influence the relevant features more directly (e.g., haemangioma on the forehead might reduce the perceived size of this part of the face). The fact that abnormalities have a large impact despite not altering the Kindchenschema indicates that additional features and attributions are clearly involved in judging cuteness.

### Effects of spatial position

In Experiment 2, abnormality type and severity was held constant, and we examined whether the location of the abnormality would be important. One possibility is that abnormalities might lead to a negative evaluation regardless of where they are located. Abnormalities robustly attract attention [15], and in our unconstrained, free-viewing task there was nothing to prevent participants from noticing and focusing on these atypical features. However, the results from Experiment 2 showed that, even when viewing the same haemangioma and the same infants, participants give lower ratings to images with abnormalities in the upper half (vs. the lower half) of the face. There was also evidence that participants give lower ratings to images with abnormalities located in the left (vs. the right) hemiface. While the size of these biases was modest and should not be overstated, they were robust in our diverse samples (Experiment 2a and 2b). The overall differences between conditions were small, particularly in the comparison between left and right positioned haemangioma, and so such results should be interpreted with caution. For example, the mean difference between the most disruptive haemangioma (in the top left) and the least disruptive one (in bottom top right) was only 0.4 on the 7-point scale. On the one hand, the effect size is small. On the other hand, even small differences in facial attractiveness may have an important impact on behaviour. The size of the position effect can be evaluated with respect to the mean differences between faces with and without abnormalities (e.g., in Experiment 1, where the addition of an abnormality led to a decrease of between 1 and 1.5 on the 7-point scale).

There are several possible explanations for the effects of spatial position. First, it might be that these results arise from the fact that participants spend more time attending to the top and (to a lesser degree, perhaps) the left side of the face. In adult faces, the eye-tracking literature shows that we pay most attention to the eye region during face processing tasks (e.g., [35]). There is also a leftward bias, both in rapid judgements to faces and in the frequency of gazes to different hemifields [39–42]. It may be, therefore, that abnormalities in these high attention areas are looked at for longer, or are seen as less discrete, than those in the lower or right halves of the face. There is only one study, to our knowledge, which shows that abnormalities in adult faces draw overt visual attention [15]. It may be that haemangioma in the lower half of the face do not succeed in overriding the bias to look at the eyes and the other core features, and that thus they seem more discrete. This is consistent with evidence that it is very difficult to avoid looking at the top half of the face [49].

In terms of the left hemifield bias, our findings are consistent with previous research linking attention to judgements in chimeric faces [39]. Specifically, participants are biased to use information from the left side of the face in gender and expression judgements, and this is partly determined by eye movement scanning. It has been proposed that face processing is lateralized to the right hemisphere, meaning that face information in the left hemifield is processed more readily [44, 45]. By this account, abnormalities on the left would activate neural structures which preferentially respond to faces and thus could be more salient in comparison to features on the right. A more general spatial bias towards objects on the left has also been reported in non-face stimuli [50, 51]. This bias, which is sometimes called “pseudoneglect”, has been linked to the right-hemisphere lateralization of attentional orienting mechanisms. The horizontal asymmetry in the present study may provide a real life example of such laterality in visual processing. We propose that haemangioma on the left contribute more to the overall impression of cuteness. It will be important to test the role of overt attention in this task in future research with eye-tracking and controlled exposure times.

There are, however, other possible explanations for why abnormality location might matter. It could be that haemangioma in the top half of the face are seen as more severe, more “important” or more “distasteful”, perhaps due to proximity to the eyes. It might also be that the overall facial configuration is impacted to a greater degree when certain features (such as the eyes) are disrupted. Such explanations are not necessarily mutually exclusive of the proposed attentional biases (after all, we may look at the top half of the face precisely because the eyes are important, e.g., for social attention:[52]). This may also account for the fact that strabismus had the biggest detrimental effect on judgements in Experiment 1, where it was just as disruptive as a cleft lip. However, it is harder to see why an abnormality on the left would be seen as more severe or distasteful than the same defect on the right.

### Conclusion

The present study shows that different abnormalities have a robust effect on aesthetic judgements of infant faces. When severity was held constant, haemangioma in high visual attention areas (the upper half of the face and the left hemiface) caused the greatest reduction to cuteness and attractiveness ratings. Because neural activation associated with the caregiving response is mediated by the level of cuteness in a face [13], abnormalities occurring in these high visual attention areas might be most detrimental for caregiving and maternal sensitivity (how well a mother can read and respond to her child’s needs).

## Supporting information

S1 Dataset Ratings data from Experiments 1 and 2. (ZIP)
